# Seasonal variations in serum 25-hydroxy vitamin D levels in a Swedish cohort

**DOI:** 10.1007/s12020-015-0548-3

**Published:** 2015-02-14

**Authors:** Eva Klingberg, Göran Oleröd, Jan Konar, Max Petzold, Ola Hammarsten

**Affiliations:** 1 Department of Rheumatology and Inflammation Research, Sahlgrenska Academy at the University of Gothenburg, Gothenburg, Sweden; 2 Department of Clinical Chemistry and Transfusion Medicine, Sahlgrenska Academy at the University of Gothenburg, Gothenburg, Sweden; 3 Centre for Applied Biostatistics, Occupational and Environmental Medicine, Sahlgrenska Academy at the University of Gothenburg, Gothenburg, Sweden; 4 Department of Rheumatology, Sahlgrenska/SU, Gröna stråket 14, 41345 Gothenburg, Sweden

**Keywords:** Vitamin D, Vitamin D deficiency, Parathyroid hormone, Ultraviolet light

## Abstract

To study seasonal inter-individual and intra-individual variations in serum 25-hydroxy vitamin D (25(OH)D) and to explore parameters associated with 25(OH)D in a healthy Swedish adult population. 540 blood donors (60 % men; mean age 41 ± 13 years) and 75 thrombocyte donors (92 % men, aged 46 ± 11 years) were included. Serum was collected during 12 months and analyzed for 25(OH)D and parathyroid hormone (S-iPTH). The blood donors answered questionnaires concerning vitamin D supplements, smoking, physical activity, sunbed use and sun holidays. Repeated serum samples were collected from the thrombocyte donors to study the intra-individual variations in S-25(OH)D. S-25(OH)D varied greatly over the year correlating with the intensity of the UV-B irradiation (*r*_S_ = 0.326; *p* < 0.001). During January–March, a S-25(OH)D level below the thresholds of 50 and 75 nmol/L was observed in 58 and 88 %, respectively, and during July–September in 11 and 50 % (*p* < 0.001). S-25(OH)D was negatively correlated with body mass index and S-iPTH, but was significantly higher in holiday makers in sunny destinations, sunbed users, non-smokers, and in the physically active. The intra-individual analyses showed a mean increase in S-25(OH)D by 8 nmol/L/month between April and August. Approximately 75 % had serum 25(OH)D values <75 nmol/L during 75 % of the year and 50 % had serum 25(OH)D <50 nmol/L during 50 % of the year. Serum 25(OH)D was strongly associated with parameters related to sun exposure, but only weakly with intake of vitamin D supplements.

## Introduction

Vitamin D is essential for the skeletal metabolism, muscle function, calcium homeostasis, and the immune system. In recent years, a growing body of observational data has demonstrated an association between poor vitamin D status and chronic illness, including autoimmune diseases, cancer, infections, diabetes, liver disease, and cardiovascular disease [1–9]. Vitamin D_3_ is a prohormone that undergoes successive hydroxylation in the liver (25-hydroxy vitamin D; 25(OH)D) and the kidneys (1, 25-hydroxy vitamin D; 1,25 (OH)_2_D). 25(OH)D is the principal form of circulating vitamin D and the metabolite which best reflects the vitamin D status [10].

Vitamin D can either be endogenously produced in the skin through the photolysis of 7-dehydocholesterol to precholecalciferol (previtamin D_3_) by ultraviolet radiation, or orally ingested through vitamin D-rich nutrients, such as fatty fish, egg yolk, certain mushrooms, or meat. However, at northern latitudes, the ultraviolet irradiance is too low to allow the photolysis of vitamin D during the winter months and the populations are dependent on the vitamin D consumed through food or supplements [11–13].

Osteomalacia occurs at low levels of 25(OH)D, usually below 25 nmol/L, but there is currently no consensus on the optimum levels of vitamin D. A serum 25(OH)D level below 50 nmol/L is considered inadequate, based on integration of bone health outcomes [14]. Adequate vitamin D status (“vitamin D sufficiency”) can also be defined as the level where the serum parathyroid hormone (PTH) is stable and does not decrease further with vitamin D supplementation, which corresponds to a serum level of 25(OH)D of around 75 nmol/L [15–17].

Low levels of 25(OH)D have been observed in significant proportions of the populations around the world and is very common among the institutionalized elderly population [15, 18–22].

The seasonal changes in vitamin D status in the healthy adult population in Sweden have not been fully evaluated. Furthermore, the proportion of the population that would need vitamin D supplementation to reach the target of a serum 25(OH)D level of 50 and 75 nmol/L, respectively, is not known. In addition, knowledge about the mean individual changes in vitamin D status over the year would allow us to predict the vitamin D status at different times of the year from a single vitamin D measurement.

The aims of the present study were, (a) to analyze the serum concentrations of 25(OH)D in healthy adult individuals during different seasons of the year; (b) to study demographic and lifestyle-related parameters associated with serum 25(OH)D; and (c) to investigate the intra-individual variation in serum 25(OH)D over time and during different seasons of the year.

## Materials and methods

### Study population

Healthy blood donors resident in Gothenburg, Sweden (57°41′N, 11°59′E), were invited to participate in the study when giving blood. A total of 540 blood donors were included consecutively, 40–60 in the middle of each month from October 2009 to September 2010.

The blood donors all stated that they were in full health and answered questionnaires regarding medication, including vitamins and complementary alternative medication, smoking habits, physical activity, use of a vegetarian diet, sunbed use, and if they had traveled to a sunny country, defined as a journey to southern latitudes below 43 °N, during the month before inclusion. Height and weight were measured and the body mass index (BMI) calculated.

To study the inter-individual seasonal variation in serum 25(OH)D, repeated serum samples were collected from thrombocyte donors during a period from April to November, 2008. Thrombocyte donors are required to be healthy and free from medication. Since thrombocytes are separated from the blood via apheresis, these donors are allowed to donate thrombocytes every 2 weeks, enabling collection of multiple samples from the same individual.

Written informed consent was obtained from all participants. The study was approved by the local Regional Ethics Committee in Gothenburg and carried out in accordance with the Helsinki declaration.

### Laboratory analyses

Serum samples were frozen at −80 °C immediately after collection and stored for up to 1 year before analysis. After thawing, the samples were all analyzed on the same day.

Serum 25(OH)D (both D_2_ and D_3_) and serum osteocalcin concentrations were analyzed with chemiluminescence immunoassay (CLIA) on a LIAISON instrument (DiaSorin Inc, Stillwater, MN, USA).

The total coefficient of variance (CV) for serum 25(OH)D was 5–6 %, with the highest variance in the lowest test range, and the functional sensitivity was 12.5 nmol/L at a CV of 8 %.

The total CV for serum osteocalcin was 4–6.5 %, with the highest variance in the lowest test range, and the functional sensitivity was 3 µg/L at a CV of 17 %.

Serum concentrations of intact parathyroid hormone (iPTH) were analyzed with CLIA on an Abbott ARCHITECT instrument (Abbott Diagnostics Division, Abbott Park, IL, USA). The total CV for iPTH ranged from 2.8 to 3.2 %. The functional sensitivity was below 5 ng/L at a CV of 20 %. The reference interval for iPTH, provided by the manufacturer, was 15–68 ng/L (percentile 2.5–97.5).

Serum calcium, albumin, and phosphate were analyzed on a Cobas instrument (Roche Molecular Diagnostics, Pleasanton, CA, USA).

### Ultraviolet radiation

Monthly sums of Commission Internationale de l’Éclairage (CIE)-weighted UV radiation (Wh/m^2^), which mimics the erythemal effect of UV radiation, were calculated for Gothenburg (57°41′N, 11°59′E) for the period October 2009 to September 2010 using the Swedish Meteorological and Hydrological Institute’s (SMHI) solar radiation model STRÅNG (www.strang.smhi.se).

### Statistics

Statistical analyses were performed using the PASW Statistics 18.0 (SPSS Inc., IBM, Chicago USA). Descriptive statistics are presented as medians and ranges and/or mean and standard deviations (SD). The *T* test was used for comparison of normally distributed variables and the Mann–Whitney *U* test for abnormally distributed variables. The *χ* ^2^ test was used to compare categorical variables. Correlations were calculated using Spearman’s correlation (*r*_s_). For dichotomous variables, yes was coded as 1 and no as 0. All tests were two-tailed and *p* < 0.05 was considered statistically significant. Linear regression was run with serum 25(OH)D as the outcome and the variables significantly associated with serum 25(OH)D in the univariate analyses as covariates (sum of CIE-weighted UV radiation during sampling month, sex, age, BMI, estrogen use, sunny holidays, sunbed use, smoking, and physical activity). A mixed model was used to analyze the data from repeated measurements.

## Results

### Demographics of the healthy subjects

A total of 540 blood donors (215 women and 325 men) with a mean age of 40.5 ± 13.0 years were included in the study. The characteristics of the blood donors are given in Table 1.

**Table 1:** Characteristics of the 540 blood donors included in the study

	Subjects *n* (%)	Median (range)	Mean ± SD
Sex			
Women	215 (39.8)		
Men	325 (60.2)		
Age (years)		41 (16, 71)	40.5 ± 13.0
Body weight (kg)		76 (36, 183)	77.6 ± 15.2
Body length (cm)		177 (155, 205)	176.6 ± 8.9
Body mass index (kg/m^2^)		24 (12, 61)	24.8 ± 4.0
Current smokers	25 (4.6)		
Subjects who had used a sunbed during the previous month	23 (4.3)		
Subjects on a sunny holiday during the previous month	36 (6.7)		
Subjects reporting a vegetarian diet	26 (4.8)		
Subjects who exercise once a week	150 (27.7)		
Subjects taking vitamin D supplements	50 (9.2)		
Female study subjects taking estrogens (contraceptives or HRT)	24/215 (11.2)		
Subjects on any medication (other than vitamin D)	62 (11.5)		
Subjects on complementary and alternative medicine	77 (14.2)		
Serum 25(OH)D (nmol/L)		60 (10, 224)	62.8 ± 26.3
Serum iPTH (ng/L)		40 (8.7, 151)	44.0 ± 18.6
Serum osteocalcin (µg/L)		14 (5.7, 39)	15.1 ± 5.4
Serum calcium (mmol/L)		2.38 (1.85, 2.82)	2.39 ± 0.13
Serum albumin (g/L)		42 (31, 50)	41.7 ± 3.1
Serum phosphate (mmol/L)		1.3 (0.7, 4.6)	1.3 ± 0.4

*25(OH)D* 25-hydroxy vitamin D, *iPTH* intact parathyroid hormone, *HRT* hormone replacement therapy

In addition, a total of 300 blood samples were collected from 75 thrombocyte donors (6 women and 69 men) with a mean age of 45.8 ± 10.9 years. Multiple blood samples, up to 11 from the same individual, were acquired.

### Seasonal variations in serum 25(OH)D: blood donors

**Fig. 1:**
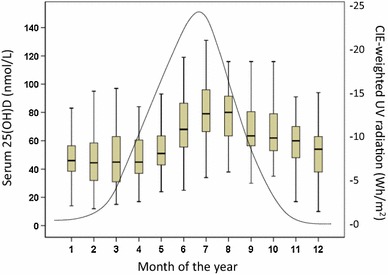
Box plot of serum 25(OH)D during the different months of the year. The values shown represent medians (*horizontal line*), interquartile ranges (*box*), and ranges of values (*whiskers*)

The CIE-weighted UV radiation increased from 0.36 Wh/m^2^ in December to 24.25 Wh/m^2^ in July and was positively correlated with serum 25(OH)D (*r*_S_ = 0.333; *p* < 0.001) Great variations were found in the mean concentrations of 25(OH)D during the different months of the year (Table 2 and Fig. 1). The mean serum concentration of 25(OH)D was 73 % higher in July (81.9 ± 26.2 nmol/L) than in February (47.4 ± 20.7 nmol/L), (*p* < 0.001). The mean serum concentration of 25(OH)D during the third quarter of the year (July to September, 77.7 ± 27.3 nmol/L) was 62 % higher than during the first quarter of the year (January–March, 47.9 ± 19.6 nmol/L), (*p* < 0.001).

**Table 2:** Serum levels of 25-hydroxy vitamin D, parathyroid hormone, calcium, and phosphate during different quarters of the year

Month	Number of subjects	25(OH)D nmol/L Mean ± SD	IPTH ng/L Mean ± SD	Calcium mmol/L Mean ± SD	Phosphate mmol/L Mean ± SD	25(OH)D <25 nmol/L *n* (%)	25(OH)D^a^ <50 nmol/L *n* (%)	25(OH)D^a^ <75 nmol/L *n* (%)
Q1	119	47.9 ± 19.6	47.2 ± 19.1	2.37 ± 0.10	1.23 ± 0.26	13 (10.9)	70 (58.8)	105 (88.2)
January	39	48.4 ± 17.4				3 (7.7)	24 (61.5)	36 (92.3)
February	40	47.4 ± 20.7				5 (12.5)	22 (55.0)	34 (85.0)
March	40	48.0 ± 21.0				5 (12.5)	24 (60.0)	35 (87.5)
Q2	140	60.8 ± 24.5	44.2 ± 18.3	2.38 ± 0.10	1.25 ± 0.24	3 (2.1)	51 (36.4)	106 (75.7)
April	40	50.3 ± 20.0				2 (5.0)	22 (55.0)	36 (90.0)
May	40	53.8 ± 17.1				1 (2.5)	19 (47.5)	33 (82.5)
June	60	72.6 ± 26.7				0 (0)	10 (16.7)	37 (61.7)
Q3	160	77.7 ± 27.3	42.4 ± 17.3	2.35 ± 0.13	1.47 ± 0.60	2 (1.3)	18 (11.3)	80 (50.0)
July	60	81.9 ± 26.2				0 (0)	5 (8.3)	27 (45.0)
August	60	80.4 ± 29.8				1 (1.7)	6 (10.0)	26 (43.3)
September	40	67.4 ± 22.7				1 (2.5)	7 (17.5)	27 (67.5)
Q4	121	60.2 ± 22.5	42.8 ± 20.1	2.47 ± 0.14	1.34 ± 0.29	7 (5.8)	36 (29.8)	97 (80.2)
October	40	69.9 ± 24.1				0 (0)	7 (17.5)	26 (65.0)
November	41	56.8 ± 18.3				4 (9.8)	11 (26.8)	37 (90.2)
December	40	54.2 ± 22.2				3 (7.5)	18 (45.0)	34 (85.0)
All^b^	540	60.9 ± 22.2				5.1 %	35.1 %	74.6 %

Serum 25-hydroxy vitamin D in relation to recommended levels during different months and quarters of the year in 540 blood donors. Numbers represent mean ± standard deviation or number (%)

*25(OH)D* 25-hydroxy vitamin D, *iPTH* intact parathyroid hormone, *Q* quarter of the year, *SD* standard deviation

^a^Cumulative lists including all subjects with S-25(OH)D below 50 and 75 nmol/L, respectively

^b^The mean of the monthly mean values. Values are adjusted for the fact that more subjects were included during the summer months

**Fig. 2:**
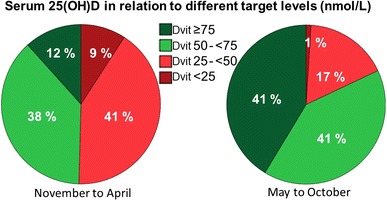
Pie-diagram of serum 25(OH)D during different seasons in relation to recommended levels

During the first quarter of the year (January–March), 58.3 % (70/120) had serum concentrations of 25(OH)D lower than 50 nmol/L, and 87.5 % (105/120) had levels lower than 75 nmol/L, compared with the third quarter of the year (July–September), when 11.3 % (18/160) had levels below 50 nmol/L and 50 % (80/160) had levels below 75 nmol/L (*p* < 0.001) (Table 2 and Fig. 2).

### Serum iPTH, osteocalcin, calcium, and phosphate and the relationship with 25(OH)D

Mean and median concentrations of serum iPTH, osteocalcin, calcium, albumin, and phosphate are shown in Table 1. Serum 25(OH)D was correlated with serum iPTH (*r*_S_ = −0.253; *p* < 0.001), serum osteocalcin (*r*_S_ = −0.087; *p* = 0.042), and serum phosphate (*r*_S_ = 0.122; *p* = 0.005), but not with serum calcium (*r*_S_ = −0.058; *p* = 0.184) or albumin (*r*_S_ = −0.058; *p* = 0.176).

There was a significant difference in serum iPTH (*p* < 0.001), but not in serum osteocalcin, between groups of subjects with different levels of serum 25(OH)D (<25; 25–49, 50–74; >75 nmol/L). A serum iPTH above the reference interval of 15–68 ng/L was observed in totally 9.1 % (*n* = 49) of the blood donors,

The relation between level of serum 25(OH)D and the percentage of subjects with a serum iPTH above 68 ng/L was as follows: <25 nmol/L 32 %, 25–49 nmol/L 11 %, 50–74 nmol/L 8 %, and >75 nmol/L 5 %.

Serum iPTH was significantly higher during the first quarter of the year (January–March) then during the third quarter (July–September) (S-iPTH 47.2 ± 19.1 vs. 42.4 ± 17.3; *p* = 0.031), whereas the opposite was observed for serum phosphate (S-phosphate 1.23 ± 0.26 vs. 1.47 ± 0.60; *p* < 0.001), which thus was lower during the first quarter. No significant differences were found in serum osteocalcin, calcium, or albumin during the different quarters of the year (Table 2).

iPTH was positively correlated with BMI (*r*_S_ = 0.193; *p* < 0.001).

### Demographic and lifestyle-related parameters in relation to concentrations of serum 25(OH)D: blood donors

The associations between serum 25-OHD and demographic and lifestyle-related parameters are shown in Table 3. The female study participants had significantly higher serum 25(OH)D levels than the men. Serum 25(OH)D was also significantly higher in sunbed users, in subjects who had visited a sunny country during the month before inclusion, in subjects doing physical exercise regularly every week, and in non-smokers compared with smokers. Among the female study subjects, the serum concentrations of 25(OH)D were significantly higher in users of estrogens (contraceptives or hormone replacement therapy) compared with non-users. Weak negative correlations were found between serum 25(OH)D and weight (*r*_S_ = −0.147; *p* = 0.001) and BMI (*r*_S_ = −0.179; *p* < 0.001), but not with age or height. No significant differences in serum 25(OH)D were found in vegetarians or between users or non-users of vitamin D supplements, complementary alternative medication, or prescribed medication (mostly contraceptives, anti-allergy, or asthma medication).

**Table 3:** Serum 25(OH)D in relation to demographic or lifestyle-related parameters in the blood donors

	Serum 25(OH)D nmol/L mean ± SD	Significance *p* value
Women versus men	65.6 ± 25.3 versus 61.0 ± 26.8	0.046
Women versus men (when sunbed users were excluded)	63.8 ± 22.9 versus 60.8 ± 27.0	0.20
Age ≤ 29 versus ≥51 years (first quartile versus forth quartile)	63.6 ± 30.2 versus 59.7 ± 21.8	0.213
BMI ≤ 22.4 versus ≥26.8 kg/m^2^ (first quartile versus forth quartile)	65.5 ± 26.3 versus 54.7 ± 22.2	<0.001
Smokers versus non-smokers	51.4 ± 26.2 versus 63.4 ± 26.2	0.031
Sunbed users versus non-users	82.2 ± 35.6 versus 62.0 ± 25.5	<0.001
Subjects on a sunny holiday during the previous month, yes versus no	86.9 ± 37.7 versus 61.1 ± 24.4	<0.001
Physically active^a^ versus non-active	64.5 ± 26.7 versus 58.4 ± 24.7	0.015
Female study subjects using estrogens^b^ versus non-users	84.0 ± 38.3 versus 63.3 ± 22.2	0.016
Vitamin D supplement users versus non-users (during the whole year)	64.7 ± 19.5 versus 62.7 ± 26.9	0.49
Vitamin D supplement users versus non-users (winter months only)	59.0 ± 21.0 versus 49.2 ± 19.4	0.01
Medication (other than vitamin D), users versus non-users	70.3 ± 34.2 versus 61.9 ± 24.9	0.065
Users of a vegetarian diet versus non-users	59.7 ± 23.1 versus 63.0 ± 26.4	0.53
Complementary and alternative medicine users versus non-users	63.3 ± 21.8 versus 62.8 ± 27.0	0.87

*25(OH)D* 25-hydroxy vitamin D, *BMI* body mass index

^a^Physically active defined as performing physical exercise regularly at least once a week

^b^estrogens were taken as contraceptives or hormone replacement therapy

In total, 50 subjects were using vitamin D supplements, mostly multivitamin preparations with daily doses of vitamin D_3_ of 5–7.5 µg (200–300 IE). When comparing the effect of vitamin D supplements during the winter and summer months, we found significantly higher serum 25(OH)D levels in vitamin D users during the winter (59.0 ± 21.0 vs. 49.2 ± 19.4 nmol/L, *p* = 0.015), but not during the summer.

We also found that physical activity (>once a week) was associated with higher serum concentrations of 25(OH)D during the summer (74.9 ± 27.5 vs. 67.2 ± 23.1; *p* = 0.026), but not during the winter.

More women than men answered that they had used a sunbed during the month before inclusion in the study (17/214 vs. 6/326, *p* = 0.001). When the sunbed users were excluded, no significant difference in serum 25(OH)D could be found between women and men.

In multiple linear regression analyses, serum 25(OH)D was independently associated with CIE-weighted UV radiation during the sampling month (*B* = 1.04; *p* < 0.001), BMI (*B* = −1.48; *p* < 0.001), estrogen use (*B* = 15.43; *p* = 0.002), and having traveled to a sunny country (*B* = 21.76; *p* < 0.001) or used a sunbed (*B* = 22.73; *p* < 0.001) during the month before the inclusion in the study (*R* ^2^ = 0.269).

### Intra-individual variations in serum 25(OH)D during different seasons of the year: results from the thrombocyte donors

**Fig. 3:**
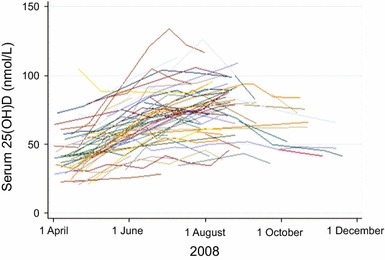
Intra-individual changes in serum 25(OH)D from April to August

The mean serum concentrations of 25(OH)D followed the intensity of the UV light irradiance in the Gothenburg area and peaked in July, whereafter they decreased. The mean increase in serum 25(OH)D from April to August was 0.268 nmol/L/day (95 % CI 0.239–0.298 nmol/L/day) or 8.0 nmol/L/month (Fig. 3). The individual minimum and maximum serum levels of 25(OH)D in the study subjects were strongly correlated (*r*_S_ = 0.684; *p* < 0.001), indicating that each individual follows his/her own curve. The intra-individual maximum change in serum 25(OH)D is shown in Table 4.

**Table 4:** Intra-individual maximum change in serum 25(OH)D in relation to target levels

Minimum levels of serum 25(OH)D % (*n*)		Maximum levels of serum 25(OH)D % (*n*)		
		25–50 nmol/L	50–75 nmol/L	>75 nmol/L
<25 nmol/L	100 % (5)	60 % (3)	20 % (1)	20 % (1)
25–50 nmol/L	100 % (33)	21 % (7)	49 % (16)	30 % (10)
50–75 nmol/L	100 % (27)	0 % (0)	33 % (9)	67 % (18)
>75 nmol/L	100 % (10)	0 % (0)	0 % (0)	100 % (10)

*25(OH)D* 25-hydroxy vitamin D

## Discussion

The present study shows that vitamin D insufficiency is common among Swedish adult healthy women and men, especially during the winter months. In total, 54 % (129/240) of the included blood donors had a serum 25(OH)D level lower than 50 nmol/L during December to May. Using the higher threshold for vitamin D insufficiency, 75 nmol/L, 75 % were found to be vitamin D insufficient during the three quarters of the year. [17]. This clearly shows that even though dairy products are supplemented with vitamin D in Sweden, the diet fails to provide an adequate amount of vitamin D during the winter months. The results thus indicate that at least half of the adult healthy population would need extra vitamin D supplementation during the winter months to reach the target level of 50 nmol/L.

Our results are in contrast to earlier studies which have shown a low prevalence of vitamin D deficiency among Scandinavian (mostly Danish and Norwegian) young adults and elderly, in comparison with the population in Mediterranean Europe [23–26]. It has been argued that this is due to high intake of fatty fish, vitamin-supplemented dairy products, and vitamin D supplements in Scandinavia. In an earlier Swedish study on women aged 61–86 years, only 19 % had a serum 25(OH)D level below 50 nmol/L during the winter; however, a high percentage, 25 %, of the participants had been on a sunny holiday during the winter [27]. Another explanation of the discrepancy could be a greater awareness of osteoporosis and the need for calcium and vitamin D intake among postmenopausal women. Sweden and the Scandinavian countries top the ranking list of ten-year probabilities of a hip fracture, which can be used as an indicator of the prevalence of osteoporosis in a country [28, 29]. The serum levels of 25(OH)D in the present study were comparable with the levels reported in an earlier study on elderly men living in the Gothenburg area [22].

Interpreting and taking action on serum 25(OH)D values analyzed during different seasons of the year may pose a problem to the clinician. In most cases, the time of the appointment is not optimally chosen to study the levels of vitamin D. The results from the current study show that a value of 25(OH)D measured in late spring can be expected to be about 35.1 ± 16.3 nmol/L higher in late summer and vice versa.

The present study shows that the mean levels of serum calcium among the participants were stable during the different seasons of the year. The maintenance of the serum calcium concentration within a narrow range is essential for physiological processes, a balance which is regulated by parathyroid hormone and vitamin D. Serum iPTH was thus significantly higher during the winter months, when serum 25(OH)D reached its nadir values. Serum phosphate was however significantly higher during the summer months, when serum 25(OH)D reached its peak values, reflecting the positive effects of 1,25 (OH)_2_D on intestinal phosphate absorption.

The main source of vitamin D is the endogenous production in the skin following sun exposure [30]. In the present study, a recent visit to a sunny country and sunbed use were strongly and independently associated with higher serum 25(OH)D levels, whereas only a weak association was found between the levels and intake of vitamin D supplements. The daily doses of vitamin D_3_ supplements reported by the participants (5-7.5 µg; 200-300 IU/day) may, however, have been too low to achieve optimum levels of vitamin D [31]. In addition, the vitamin D supplements may only have been taken sporadically. Our results are supported by a recent Swedish study on middle-aged female primary care patients, which reported a strong association between sunny holidays and vitamin D status during the winter, but no association between serum 25(OH)D levels and intake of vitamin D by food or supplements. The prevalence of serum 25(OH)D levels below 50 nmol/L was comparable to our results, 50 % [32].

We found that the use of hormone replacement therapy or oral contraceptives was independently associated with a higher serum 25(OH)D. An explanation for this could be that estrogens stimulate the synthesis of vitamin D-binding protein (DBP) [33]. One earlier study also found increased levels of vitamin D metabolites and DBP in women using oral contraceptives [34]. Increased levels of DBP have also been found in pregnant women and in postmenopausal women using hormone replacement therapy [35].

The association between low serum 25(OH)D and high BMI found in the present study is supported by several previous studies [36–38]. Vitamin D deficiency in obese individuals may be caused by storage of 25(OH)D in the adipose tissue. Other proposed mechanisms are high expression of the vitamin D receptor (VDR) in adipose tissue and vitamin D possibly playing a role in the pathogenesis of the metabolic syndrome. [5] We found lower levels of serum 25(OH)D in smokers than in non-smokers. Earlier studies have yielded conflicting results, showing lower, unchanged, or higher levels of 25(OH)D in serum among smokers [39–41].

Study subjects performing exercise regularly, at least once a week, had higher serum concentrations of 25(OH)D than the physically inactive during the summer period, but not during the winter. Our interpretation of this result is that physical activity during the summer is associated with out-door activities.

Numerous studies have linked chronic illnesses with hypovitaminosis D. To prove whether a poor vitamin D status is the cause or the result of the illness is however a challenge, since there are many possible confounding factors. The present study puts emphasis on the importance of controlling for season in studies conducted at northern latitudes. Many chronic illnesses are associated with a decreased physical function, less out-door activity and consequently less UV-B exposure. Liver and bowel diseases can in addition lead to malabsorption of vitamin D. Chronic illness is also often associated with a poorer socioeconomic status, lower educational level, and reduced economic resources to spend on sun holidays, multivitamins, and vitamin D-rich nutrients such as fish and shellfish.

Limitations of the present study were the lack of information regarding the dietary intake of vitamin D of the participants. The absolute majority of the participants were Caucasians, but more detailed information about skin type or color was not obtained. In addition, blood and thrombocyte donors are selected populations and the thrombocyte donors were mostly male. As a result, the study may not be representative of the general Swedish population.

## Conclusions

Vitamin D insufficiency is common in the Swedish healthy adult population. We found that approximately 50 % of the study population had serum concentrations of 25(OH)D below 50 nmol/L during at least half of the year, and that 75 % had concentrations below 75 nmol/L during 75 % of the year. Furthermore, the study indicates that it is possible to extrapolate individual seasonal variations in 25-OHD levels from a single serum sample. The serum 25(OH)D concentration was strongly associated with exposure to UV light but only weakly with intake of vitamin D supplements.
